# Colonial green algae in the Cambrian plankton

**DOI:** 10.1098/rspb.2023.1882

**Published:** 2023-10-25

**Authors:** Thomas H. P. Harvey

**Affiliations:** Centre for Palaeobiology and Biosphere Evolution, School of Geography, Geology and the Environment, University of Leicester, University Road, Leicester LE1 7RH, UK

**Keywords:** colonial, algae, Cambrian, plankton, phytoplankton, Cambrian explosion

## Abstract

The fossil record indicates a major turnover in marine phytoplankton across the Ediacaran–Cambrian transition, coincident with the rise of animal-rich ecosystems. However, the diversity, affinities and ecologies of Cambrian phytoplankton are poorly understood, leaving unclear the role of animal interactions and the drivers of diversification. New exceptionally preserved acritarchs (problematic organic-walled microfossils) from the late early Cambrian (around 510 Ma) reveal colonial organization characterized by rings and plates of interconnected, geometrically arranged cells. The assemblage exhibits a wide but gradational variation in cell size, ornamentation and intercell connection, interpreted as representing one or more species with determinate (coenobial) colony formation via cell division, aggregation and growth by cell expansion. An equivalent strategy is known only among green algae, specifically chlorophycean chlorophytes. The fossils differ in detail from modern freshwater examples and apparently represent an earlier convergent radiation in marine settings. Known trade-offs between sinking risk and predator avoidance in colonial phytoplankton point to adaptations triggered by intensifying grazing pressure during a Cambrian metazoan invasion of the water column. The new fossils reveal that not all small acritarchs are unicellular resting cysts, and support an early Palaeozoic prominence of green algal phytoplankton as predicted by molecular biomarkers.

## 1. Introduction

The phytoplankton underpins marine food webs, global biogeochemical cycles and climate patterns, as it must have done for much of Earth history. Nevertheless, the fossil record shows major shifts in the composition and structuring of the phytoplankton, offering insights into the long-term drivers of planktic ecosystems. The dominant modern groups—the dinoflagellates, coccolithophores and diatoms—have good fossil records consisting of recalcitrant organic-walled cysts and calcareous or siliceous armour, respectively; they diversified in a Mesozoic ‘revolution’ parallel to that of marine metazoans [1,2]. Another fundamental revolution is suggested to have occurred during the Proterozoic–Phanerozoic transition, with metazoan grazing and filtration transforming a poorly ventilated, turbid water column dominated by cyanobacterial picoplankton into a ‘clear-water’ system with larger eukaryotic algae, with knock-on effects for biogeochemical cycling [3]. However, details of this transition are obscured because most candidate fossil phytoplankton through this interval belong to the acritarchs, an artificial, polyphyletic grouping of organic-walled microfossils of unknown biological affinity [4].

The acritarch record shows a major shift at the base of the Cambrian with the appearance and diversification of acanthomorphs of small size (a few tens of micrometres) and often elaborate ornamentation [5–7]. They contrast with the unusually large Ediacaran forms that mostly disappear from the record by the Cambrian, and are variously interpreted as benthic heterotrophs [6,8] or giant phytoplankton [9]. Either way, the appearance of small acanthomorphs in the early Cambrian can be viewed as the advent of a Phanerozoic-type phytoplankton dominated by eukaryotic algae and shaped by novel metazoan grazing pressure [8].

A prevailing assumption is that most acritarchs represent the resting cysts of unicellular, phytoplankic organisms [6,8,10,11], with interpretations of morphology, ecology and diversity often guided by comparisons with dinoflagellates [12–14]. Characteristic dinoflagellate biomarkers (dinosteranes) have been reported from the Cambrian and earlier intervals [15], but given the absence of convincing fossil dinoflagellates prior to the Mesozoic, and the phylogenetically derived condition of dinosterol synthesis in the group [16], an earlier non-dinoflagellate producer seems likely [17]. Instead, Palaeozoic acritarchs may largely belong to the green algae [1,18]. Bulk-rock biomarker analysis suggests that green algae were the predominant eukaryotic phytoplankton in the Ediacaran and Palaeozoic [19], consistent with molecular phylogenetic reconstructions of green algal history [20]. For the vast majority of acritarchs, however, direct fossil evidence for affinity and ecology is lacking. Some former acritarchs have been reclassified as the phycoma stages of green algal prasinophytes [21], and a case has been made for interpreting certain Cambrian (and Ediacaran) acritarchs as the life-history stages of unicellular green algae [9,17,22]. Recently, contrasting interpretations of colonial lifestyles have been mooted for Cambrian acritarchs preserved in monotypic clusters [23], although non-biotic means of cluster formation cannot be ruled out [23,24].

Here, I report an exceptionally well-preserved assemblage of organic walled microfossils from the lower Cambrian Forteau Formation (Stage 4; Canada) in which morphologically diverse ‘acritarchs’ occur in biologically interconnected clusters. They are definitively not dinoflagellates, or prasinophytes, or any sort of unicellular resting cyst. Instead, comparisons with modern analogues identify them as vegetative stages of planktic green algae. The implications are explored for acritarch palaeobiology and the nature of the Cambrian plankton.

## 2. Material and methods

The Forteau Formation of Newfoundland and Labrador, Canada, consists of carbonates and siliciclastics laid down on the Laurentian margin during a deepening–shallowing cycle in a nearshore to offshore shelf setting [25]. In the sampled region, a nearshore carbonate-rich succession with local development of archaeocyath patch reefs and ooidal limestones is overlain by transgressive mudstones and nodular carbonates (Middle Shale Member), above which are coarser sediments from a prograding barrier shoal complex [25]. The formation is dated to the regional Dyeran Stage based on trilobites including olenellids [25], equivalent to international Cambrian Series 2, Stage 4, *ca* 506–514 Ma [26].

Horizons targeted for sampling were fine-grained, undeformed mudstones from the Middle Shale Member exposed at three localities: Mount St. Margaret Quarry and ‘Ten Mile Lake Quarry’ (MSMQ and TMLQ, western Newfoundland), and L'Anse-Au-Loup Quarry, southern Labrador (LALQ) (see electronic supplementary material, table S1 for details). The sampled intervals were laid down in intermediate relative water depths in an open shelf setting, below storm wave-base but with occasional thin storm-deposited bioclastic grainstones, and stratigraphically close to the interpreted maximum flooding surface [25]. The associated marine fauna includes trilobites, hyolithids, echinoderms, brachiopods, sponges and *Salterella* [25,27]. Samples of up to 50 g were processed using standard palynological techniques [28] or a low-manipulation hydrofluoric acid extraction optimized for small carbonaceous fossils (SCFs) [29]. Of the 59 recovered colonies, 46 occur on palynological strew slides among thousands of acritarch specimens including *Skiagia*, *Navifusa*, *Retisphaeridium*, *Multiplicisphaeridium*, *Comasphaeridium*, leiospherids and *Gloeocapsomorpha*-like aggregations. Thirteen specimens were hand-picked from residues with SCFs, including sponge spicules, priapulid scalids and *Wiwaxia* sclerites, and mounted individually on glass slides using a pipette. All specimens are figured. Images are transmitted light micrographs using differential interference contrast, with focal planes merged digitally. Specimens are reposited at the Geological Survey of Canada (GSC), Ottawa, Ontario, Canada, with numbers indicated in the figure captions, and England Finder coordinates listed in electronic supplementary material, table S2.

## 3. Results

### (a) Fossil description

**Figure 1.**
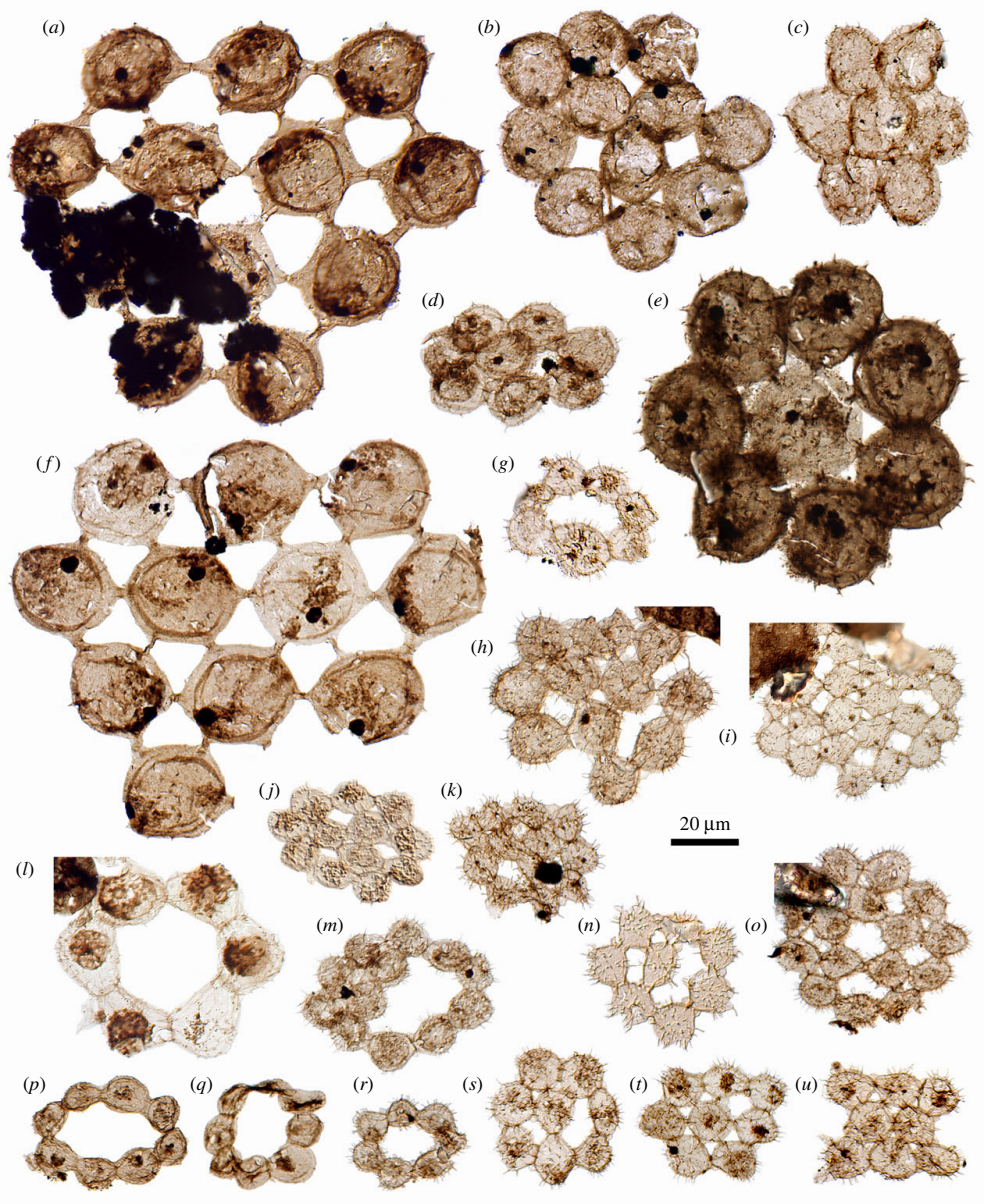
Coenobial microfossils from the lower Cambrian Forteau Formation, including strut-form colonies (*a,f*), plate-form colonies (*b–e, h–k, m–o, s–u*) and ring-form colonies (*g,l,p–r*). Specimen repository numbers GSC 143279–143299. Scale bar = 20 µm.

**Figure 2.**
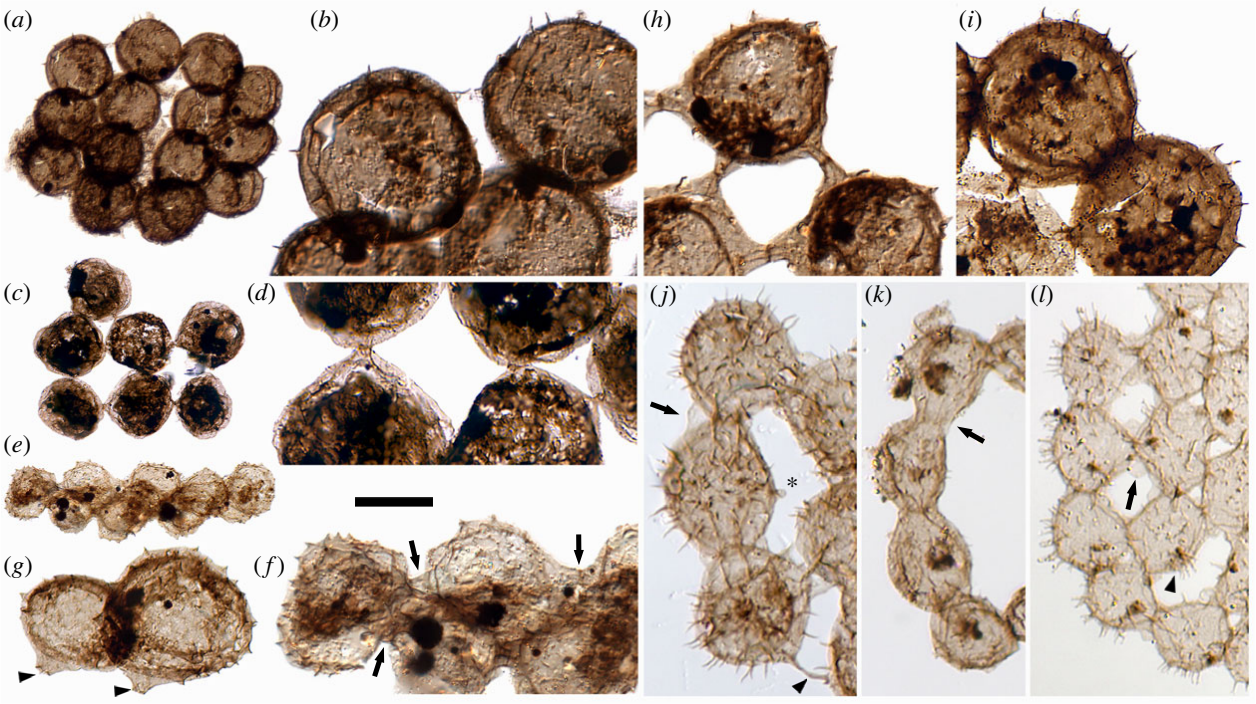
Intercell attachment structures in coenobial microfossils from the lower Cambrian Forteau Formation. (*a*) Detail in (*b*) a large plate-form colony with cryptic attachment structures (GSC 143300); (*c*) detail in (*d*) a fragment of a large fenestrate colony with short struts (GSC 143301); (*e*) detail in (*f*) a strut-form colony preserved in lateral compression, with struts arrowed (GSC 143302); (*g*) a group of two or more cells with broken struts (arrowheads), presumably detached from a colony (GSC 143303); (*h*) detail of a strut-form colony (figure 1*a*) (GSC 143279); (*i*) detail of a plate-form colony with short radial struts and peripheral plaques (figure 1*e*) (GSC 143283); (*j*) detail of a plate-form colony (figure 1*h*) with plaque connections and locally developed lobes (arrow), a strand connection (arrowhead) and a possible undeveloped connection site (asterisk) (GSC 143286); (*k*) detail of a ring colony (figure 1*p*) with locally striated plaque connections (arrow) (GSC 143294); (*l*) detail of a plate-form colony (figure 1*i*) with plaque connections and locally developed lobes (arrow) and processes preferentially developed on unconnected cell regions (arrowhead) (GSC 143287). Scale bar = 40 µm for (*a,c,e*); 20 µm for (*g*); 15 µm for (*b,d,f,h,i*); 10 µm for (*j,k,l*).

The colonies (*n* = 59) each consist of a single layer of cells arranged in a ring or plate, but with marked variation in the size, shape, number and arrangement of constituent cells (figures 1 and 2; electronic supplementary material, figures S1 and S2; numerical data listed in electronic supplementary material, table S2 and plotted in electronic supplementary material, figure S3). Intact colonies are recognized by symmetrical cell arrangements without broken attachment structures on the perimeter. Colonies vary in maximum dimension from 35–135 µm (mean = 63 µm, s.d. = 29 µm, measuring from 42 specimens and accounting for slight oblique deformation where present, but excluding colonies with substantial taphonomic distortion). Cell counts range from six (figure 1*l*) to more than 23 (figure 1*i*), with many specimens having seven, eight or twelve cells (*n* = 9, 10 and 7, respectively, out of 33 intact colonies, including two reconstructed on the evidence of broken connection sites, figure 1*b,f*). Ring-form colonies (*n* = 7) have six, seven or eight cells (figure 1*g,l,p,q,r*). Plate-form colonies are variously compact (close-packed) or fenestrate, with outlines ranging from triangular (figure 1*b,h,j,k*), four-sided (figures 1*u* and 2*a*) or rounded (figure 1*e,n,o*). They have either a single central cell (figure 1*c,d,e*) or a cluster of three to five cells surrounded by more or less concentric rings (figure 1*j,k,o*), or a more orthogonal packing arrangement (figure 2*a,c*).

Cell size (i.e. the vesicle/cell ‘body’ diameter, excluding processes) varies among colonies from 9 to 35 µm (mean = 18 µm, s.d. = 7.5 µm, from 59 colonies, averaging four or more undeformed measurements per colony). However, cell size is notably consistent within each colony, allowing for slight taphonomic distortion; a rare exception is a 7-cell ring with one cell wider by *ca* 30% (figure 1*g*). Cells (minus processes) are rounded to subrounded in outline and nearly spherical in large specimens (figure 2*e,f*), ranging to ovoidal or subtriangular in some smaller specimens (figure 1*j,n*), with a variety of specialized attachment structures (figure 2). The most conspicuous are long struts that number between two and six depending on the position in the colony (figure 1*a,f*), each connecting to another from a neighbouring cell with a transverse division midway (figure 2*h*), thus forming prominent fenestrae. Struts arise from a conical expansion/envelope of cell wall, revealed in specimens preserved in oblique or lateral compression (figure 2*e–g* and electronic supplementary material, figure S2*a*), giving the sense in plan view of a localized double wall and an equatorial ‘flange’ where bases of adjacent struts coalesce (figures 1*a,f* and 2*h*). Struts may be relatively short and unobtrusive, and fenestrae smaller or absent (figures 1*e* and 2*i*). In colonies without struts, cells can be connected via subtle disc-shaped junctions (plaques; figures 1*b* and 2*a*) or broader ‘reinforced’ regions of cell wall reconstructed as ring-shaped collars, sometimes with prominent striae/ridges (figure 2*j,k*) extending locally into rounded lobes (figure 2*j,l*). Attachment structures can vary within a colony: one 8-cell plate has short struts connecting to the central cell but broad plaques between peripheral cells (figures 1*e* and 2*i*), and two plaque-bearing specimens have singular contact points formed by narrow strands (figure 2*j* and electronic supplementary material, figure S2*e*). The number of attachment sites on interior cells varies with spacing, thus round-plate 8-cell colonies can have either three, four, six or seven connections to the central cell (compare figure 1*e,n,s,t*). Where attachment structures are indistinct, identification of a colonial habit is supported by the stretched or truncated appearance of cells along their connecting margins (e.g. figure 1*b,i,j*) and/or a geometric cell arrangement (figure 2*a*).

All but three specimens are ornamented with processes that range from slender hairs (figure 2*j,l*) to broad-based, pointed-tipped, thorn-like spines (figure 2*b,f,g,h,i*), all appearing solid rather than hollow. Processes range from 0.5 to 4.0 µm in length, but are notably consistent in size and morphology within a colony. They can be as long in small cells as in large cells, with lengths representing 5–28% of vesicle diameter in cells measuring 10 µm or less (compare figure 1*k,q*; plots in electronic supplementary material, figure S3*e,f*). By contrast, larger cells have spines rather than hairs, with lengths representing 5–10% of vesicle diameter in the four largest intact colonies (figures 1*a,e,f* and 2*b*). Processes are more strongly developed on unconnected regions of the cell (figure 2*g* and electronic supplementary material, figure S2*a*), extending out from the edge of the colony and sometimes into fenestrae (figures 1*h–k,n,o,s–u* and 2*g*,*j*,*l*). As a corollary, the distribution of processes can help distinguish intact colonies from fragments regardless of symmetry, because broken attachment sites will lack hairs or spines. Thus, a singular 4-cell specimen is identified as a fragment because one of its cells is smooth (electronic supplementary material, figure S1*j*), whereas a specimen with unusual 3–2–3 cell arrangement bears processes around its whole perimeter, suggesting it is intact (figure 1*u*).

Cells in the assemblage vary in wall thickness and the distribution of internal structures (inclusions). Internal subcircular patches of granular or diffuse material *ca* 5–15 µm in diameter occur in 13 specimens and can vary among cells within a colony (e.g. figure 1*a,f,l,o*). Smaller internal bodies, typically subcircular, opaque and 2–4 µm in diameter, occur in 17 specimens, consistently with one per cell (e.g. figures 1*a,f,i* and 2*l*). Some cells contain both types of inclusion (figure 1*a,f*). Occasional irregular tears probably represent post-mortem damage (e.g. figure 1*a,b,f*), but one colony (figure 1*n*) has consistent radial openings on several marginal cells that could represent dehiscence structures; notably this specimen lacks preserved cell contents.

There is no simple relationship between colony size, cell number and cell size. In general the largest colonies have the largest cells and struts only occur in large specimens, whereas ring-form colonies are comparatively small (figure 1 and electronic supplementary material, S3*c,d*). Nevertheless, equivalent cell arrangements can be expressed at different scales: hexagonally arranged 12-cell colonies can have large struts and fenestrae (figure 1*a,f*) or more closely packed cells (figure 1*b,h,j,k*), and the rounded star-shaped plates of seven or eight cells range from 40 to 95 µm in diameter (figure 1*c* and electronic supplementary material, figures S1 and S2). Given the commonalities in form and distribution of ornamenting hairs and spines, the spectrum of cell and colony sizes, and the intergrading colony arrangements and intercell attachments, it is difficult to distinguish separate sub-groups (electronic supplementary material, figure S3). Instead, the assemblage seems to represent a single, highly variable species, or a few related species with a common underlying biology.

## 4. Discussion

### (a) Overlaps with described fossil taxa

The Forteau colonies with intercell struts are apparently unique in the fossil record. However, some of the ring-form colonies (e.g. figure 1*p,q*) closely resemble in size and shape the rare Silurian palynomorph *Kahfia* [30,31], which is known from 8-cell and 16-cell rings with circular to ovoid cell outlines and thickened, striated intercell connections. The Forteau rings differ in their distribution of cell numbers, and in having spinose rather than granulate/papillate ornamentation, but could be accommodated in *Kahfia* with a slightly emended diagnosis.

The compact plate-form colonies with four-sided outlines resemble the Middle Ordovician to lower Silurian *Tapetisphaerites* [31], a genus erected to distinguish genuine colonies of four, eight and 16 tightly connected cells among specimens previously assigned to *Synsphaeridium* [10,30]. The type material includes variants with a central square of four cells (cf. figure 2*a*) and one with 3–2–3 cell packing (cf. figure 1*u*), and specimens with an ‘echinate’ ornamentation of 3 µm spines, preferentially developed on outer margins of the colony (pl. 1 figs 1,2, 4–8 of [31]). The Forteau specimens differ in having a greater variety of cell numbers and arrangements (with no unambiguous 4-cell colonies), a larger maximum cell size (beyond 7–13 µm) and small fenestrae even in the closest-packed arrangements, rather than tight polygonal cell junctions—but a relationship with *Tapetisphaerites* should be considered likely.

The Forteau assemblage presents a taxonomic conundrum: alongside the specimens comparable to *Kahfia* and *Tapetisphaerites* are intergrading forms, including close-packed colonies with circular outlines (figure 1*c*) and ring-shaped colonies partly infilled with additional cells (figure 1*m* and electronic supplementary material, figure S2*p*). The strut-form colonies represent a further morphological end-member. Distinguishing between a single, highly variable species and a collection of closely related taxa will require further data on co-occurrence. For now, the Forteau fossils are left in open nomenclature and referred to as strut-form colonies, ring-form (*Kahfia*-like) colonies, and plate-form (including *Tapetisphaerites*-like) colonies.

### (b) Inferred mode of colony formation

**Figure 3.**
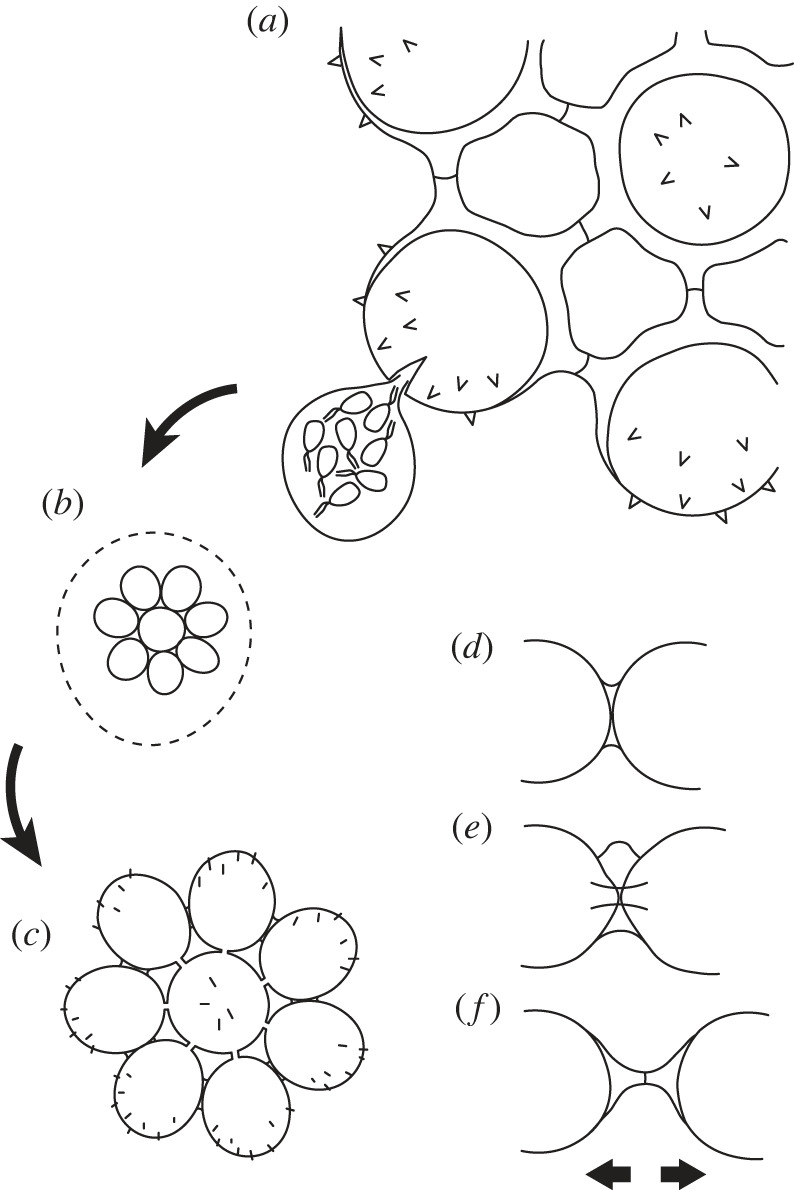
Reconstructed mode of colony formation, based on inferences from fossil morphology and variability, plus interpretative details from the asexual life-cycle of modern *Pediastrum* (after refs [32,33]). (*a*) Part of the parent coenobium with one cell splitting to extrude a vesicle along with eight clonal zoospores (extruded vesicle and biflagellate zoospore condition based on *Pediastrum*); (*b*) the degrading vesicle in which the zoospores have jostled together tightly in a plane, formed connections and then lost their flagella; (*c*) the released daughter coenobium that has developed surface ornamentation and differentiated intercell connections, and that may grow via synchronous cell expansion. Here a 12-cell strut-form colony (compare figure 1*a*) gives rise to an 8-cell rounded plate with short radial struts and peripheral plaques (compare figure 1*e*) to illustrate the hypothesis that the various colony forms can arise from one another through a highly plastic developmental process. (*d–f*) Intergrading styles of intercell connection ranging from adhesive plaques (*d*), to striated and lobed collars associated with ‘stretched’ cell outlines (*e*), to conical-based struts (*f*) that push the cells apart as they extend (arrows).

In spite of their marked variation, the Forteau colonies show evidence for a common mode of formation, reconstructed in figure 3 based on the following inferences (with interpretative details from modern analogues justified below). Within each colony, the similarity of constituent cells suggests a clonal origin and a determinate cell number that is fixed upon formation (figure 3*a–c*) rather than colony growth via sequential cell capture or cell division. The geometric and often close-packed arrangements suggest that cells arranged themselves in a single layer within a confining structure (figure 3*b*); ring colonies imply circumferential assembly. Where cells touched their neighbours, adherent regions of cell wall formed, resulting in variable numbers of contact points for interior cells and an absence of attachment structures other than at ‘realized’ contact points (figure 3*c*). Variable elaboration of attachment points produced a spectrum ranging from inconspicuous plaques, to ring-shaped collars with ridges or lobes, to increasingly elongate, conical-based struts that pushed cells apart as they grew (figure 3*d–f*). Finer spines or hairs developed preferentially on unconnected cell surfaces, outwards from the colony edge and locally into fenestrae. The consistent cell size within a colony implies that growth must have been achieved by synchronous cell expansion to prevent colony distortion.

### (c) Comparisons with extant taxa

The fundamental features of colony formation and growth inferred for the Forteau assemblage are shared with several groups of modern green algae (Chlorophyta). In particular, three groups of algae within the chlorophycean chlorophytes—Volvocales (= Chlamydomonadales), Scenedesmaceae and Hydrodictyaceae—characteristically form clonal colonies of fixed cell number known as coenobia. This strategy has evolved at least twice in volvocines [34] and independently at least once in non-volvocines, which are nested phylogenetically among unicellular species [35]. However, all share a basic pattern of colony (coenobium) formation in which successive mitotic divisions of a parent cell produce daughter cells that aggregate into a more or less regular arrangement [32] (cf. figure 3). Once the daughter colony becomes free from the parent cell (or derived vesicle), the constituent cells can grow by expansion or change shape, but they cannot multiply further. The process repeats to form a new daughter colony from each cell (or gametes where there is a switch to a sexual generation).

The modern groups differ from one another in detail, and the Forteau algae are furthermore distinct in their precise combination of characters. In having rounded cell outlines, the fossils most resemble the volvocine genus *Gonium*, which constructs plate-form coenobia of typically eight or 16 cells, either close-packed or separated by strands and thus with strikingly similar colony arrangements to the fenestrate fossils [36]. However, in *Gonium* and related volvocines, the cells are held together by cytoplasmic ‘bridges’ reinforced or replaced with extracellular matrix [37], i.e. attachment structures lacking the potential to preserve as rigid struts like those in the Forteau fossils.

By contrast, coenobial green algae with tough cell walls made from decay-resistant biomacromolecules (notably algaenans) occur among scenedesmaceans and hydrodictyaeans [38]. Various scenedesmacean genera (and similar taxa of disputed position) form coenobia with preferential development of ornamenting spines and bristles on the outer margin of the coenobium (e.g. *Scenedesmus*, *Tetrastrum*, *Crucigenia*) [39,40], and this ornamentation can show marked intraspecific variability [32]. The intercell connections can be robust, sometimes with the appearance of ‘stretched’ cell wall extending between adjacent cells, or thickened circular plaques of connecting cell wall, e.g. in *Scenedesmus* [39] and *Coelastrum* [41,42]. Notably, the development in *Coelastrum* of these plaques at sites that are not in contact with adjacent cells [42] suggests presumptive sites for connection, rather than the connection sites being specified at the moment of contact, as can be inferred for the fossils. Furthermore, cell arrangements in scenedesmacean coenobia tend to be dissimilar to the discoidal plates in the Forteau fossils and other living groups, instead consisting of linear rows of cells (*Scenedesmus*) or square, cubic or ball-like aggregations (e.g. *Crucigenia* and *Coelastrum*, respectively), presumably a constraint on colony architecture arising from having non-motile daughter cells (autospores) [41,43].

Overall, the best comparisons among living groups are with hydrodictyaceans, especially the planar, discoidal plates of ‘*Pediastrum*’ (a genus of disputed monophyly [44]). These are closely comparable to the fossils in colony size, cell number (often 4–32 cells; [32]) and in having concentric, close-packed or fenestrate cell arrangements [45–47] or rings of four and eight cells [46]. In *Pediastrum*, the coenobium is formed by flagellate zoospores that ‘swarm’ within a vesicle derived from the parent cell that is extruded through a slit in the cell wall, subsequently breaking down to release the daughter colony [45] (cf. figure 3*a–c*). The vesicle is somewhat confining but is three-dimensional so cannot fully constrain the planar colony arrangement, which must also rely on zoospore behaviour and subcellular labelling [46,47]. In contrast to the soft, motile volvocines, the cells then lose their flagella and develop a thick, decay-resistant wall, which in *Pediastrum* is especially tough, consisting of algaenans [38] and reinforced by silica [48]. The colony cells grow dramatically by expansion: 16-cell coenobia of *Pediastrum boryanum* can double or triple in diameter from initial formation to reproductive maturity (from *ca* 20 to 60 µm), taking just 50 h in favourable conditions [33]. Even more pronounced is growth in *Hydrodictyon*, where zoospores of under 10 µm grow to mature cells of several millimetres or even centimetres in length [49]. The wide size range of Forteau cells compared to many Cambrian acritarch genera [24] is thus consistent with hydrodictyacean-type growth.

For all their similarities to the fossils, modern hydrodictyaceans have cells of diverse, sometimes complex shape that are often taxon-specific, in contrast to the more or less rounded cell ‘bodies’ in the variously shaped Forteau colonies. A common elaboration of cell and colony outline in *Pediastrum* arises through the elongation of ‘horns’ from unconnected sites, forming cog-wheel-shaped colonies [46]. By contrast, the Forteau fossils exhibit strut formation only at contact points, whereas unattached sites develop minute spines or hairs, revealing a fundamentally distinct process for modifying cell outline.

In sum, coenobial green algae offer an extensive suite of comparisons to the fossil colonies. Other forms of aggregated cells occur among protists and multicellular organisms, whether as colonies or within sporangia (for example), but none share the properties of interconnected cells in planar geometric arrangement. A surprising distinction with modern coenobia, however, is in the observed range of cell numbers. Modern coenobial algae under ideal conditions construct colonies of 2*^n^* cells, from *n* number of successive mitotic divisions. Research into teratological forms to establish the processes of colony formation has documented disordered colonies lacking neat geometries, outlines and/or symmetry for *Gonium* [50], *Pediastrum* [45–47] and *Hydrodictyon* [49]. Unusual cell numbers (non-2*^n^*) are apparently less common. *Gonium* colonies with ‘gaps’ can result from the death or escape of some individual cells [50], which might be the case for certain Forteau colonies (figure 1*h,m* and electronic supplementary material, figure S1l), if not damaged post-mortem. *Gonium* and *Pediastrum* colonies with an unusual number of unequally sized cells are generally attributed to incomplete cell division [47,51], which in *Pediastrum* can be induced experimentally [52]; the Forteau specimen with obviously unequal cell size (figure 1*g*) may fall into this category. Harder to explain are the frequently encountered 7-cell colonies (*n* = 9; e.g. figure 1*c,r*) and those with 12 (not 16) equally sized cells in perfect geometric arrangement (*n* = 7; e.g. figure 1*a,f,j,k*). Clearly the inferred mode of colony formation—close swarming of zoospores followed by attachment at contact points (rather than at presumptive sites)—has the ability to produce an ordered, symmetrical colony from a wide range of cell numbers. Conceivably, the unusual numbers of cells arose through a failure to extrude some zoospores along with the vesicle from the parent cell (an observed step in *Pediastum* [52]) or, hypothetically, from the death of one of eight cells after the third round of cell division (producing seven cells), or of one of four cells after the second round (leaving three to produce six and then twelve). Less parsimoniously, the process may have involved partitioning of zoospores from a parent cell into more than one daughter colony (unknown in modern coenobia) or differentiating some cells for roles other than colony formation (today restricted to the germ–soma division in giant coenobia of *Volvox* [37]).

### (d) The fossil record of coenobial green algae

The Forteau assemblage provides the first unambiguous evidence of colony-forming, interconnected ‘acritarchs’ in the Cambrian fossil record. By comparison with modern analogues, these colonies were vegetative and coenobial, and likely belonged to chlorophycean chlorophytes, where this strategy has arisen several times. Given their distinctive mode of cell attachment and colony formation, the Forteau fossils are not easily assigned to any modern coenobial group at a lower taxonomic level (Hydrodictyaceae, Scenedesmaceae or Volvocales) and they perhaps represent an independent green algal exploration of coenobial habit. This hypothesis is supported by a review of the fossil record and the inferred histories of modern groups, which furthermore suggests long-term shifts in the ecologies and taxonomic composition of coenobial green algae.

The comparatively soft colonies of volvocines are not preserved in the palynological record, and putative spheroidal colonies in Devonian cherts [53] lack definitive characteristics. In any case, molecular clocks predict a Mesozoic diversification for the multicellular members of this lineage [54]. By contrast, fossils attributed to hydrodictyaceans and scenedesmaceans are widespread but sporadically distributed as palynomorphs, with the richest record being from Mesozoic and Cenozoic non-marine deposits [10,32,43]. Forms very similar to modern *Pediastrum* and *Scenedesmus* date back to the Jurassic, and *Tetrastrum* to the Late Triassic [55]. These Mesozoic occurrences are consistent with a predicted molecular divergence date for *Pediastrum* and *Scenedesmus* ranging from mid-Palaeozoic to Mesozoic [56].

Earlier coenobial fossils are more difficult to relate to living taxa, obscuring the origins of the modern families. Prominent in some non-marine and marginal settings in the Permian and Triassic are sheet-like arrangements that are variously compact or net-like, orthogonal or diagonal, plus associated planar star-shaped forms [57,58]. Sheets of elongate or squarish cells extend back to the Devonian [59] and Ordovician [60,61]. Like the Triassic examples, these tend to be assigned to Hydrodictyaceae or Scenedesmaceae where the cell arrangement is more linear [55]. Four-cell constructions include *Quadrisporites*, formerly regarded as a cryptospore of possible land-plant origin, but now interpreted as an alga where there is evidence of dehiscence structures [62]. There are also six Palaeozoic genera of small, planar or three-dimensional colonies assigned to Hydrodictyaceae [55,63], including the compact three-dimensional 7-cell *Ericanthea* (Ordovician) [64,65] and star-like colonies of elongate cells including the 4-cell ring-form *Deflandrastrum* (Silurian) [66] and the 4- or 8-cell *Speculaforma* (Ordovician/Silurian boundary) [63]. Ring-form colonies of six or seven rounded cells (i.e. unexpected cell numbers) with peripheral rounded openings occur in the Devonian [67,68]. Finally, as noted above, *Kahfia* (Silurian) and *Tapetisphaerites* (Ordovician to Silurian) [31] resemble components of the Forteau assemblage.

Notably, all of the older Palaeozoic (pre-Carboniferous) records are from marine deposits, apparently at odds with the freshwater (occasionally brackish) habitats of modern coenobial algae [31,68]. Modern *Pediastrum* can be transported far out to sea and recovered from marine sediments [10,55], which is perhaps unsurprising given its ability to convert vegetative colonies to resistant ‘resting cells’ aided by its tough cell walls [48]. By extension, fossil coenobia in marine deposits are inferred to be transported there [55], but whether this holds true in the Palaeozoic is questionable. Patterns of co-occurrence with plant material support a fluvial input for some assemblages, notably those with extensive sheets of orthogonally arranged cells [59,61]. Reworking of palynomorphs can potentially produce a mixed signal, and enhanced preservation in marine deposits has been mooted [68]. Nevertheless, the Forteau assemblage contains numerous well-preserved colonies of delicate construction in an offshore setting with fully marine invertebrates. Previous reports of *Kahfia* and *Tapetisphaerites* are likewise from marine deposits [31]. Given the extinct combination of characters with no stratigraphic continuity to comparable extant coenobia and the molecular clock predictions of later divergence times within Hydrodictyaceae and Volvocales, the Forteau fossils more parsimoniously represent an independent marine radiation of coenobial green algae in the early Palaeozoic. If instead the Forteau coenobia were non-marine, this is the first Cambrian assemblage from such a setting. No coeval freshwater deposits exist for a direct comparison, but the general absence of reported coenobia from lower Palaeozoic non-marine deposits is striking.

The Precambrian fossil record is rich in clustered and ordered cells, but mostly from actively proliferating colonies or those lacking intercell attachments [69]. A notable exception is the mid-Neoproterozoic *Palaeastrum*, which occurs as closed-cylindrical colonies of hundreds of cells (like modern *Hydrodictyon*) [69] and with circular attachment plaques reminiscent of those in *Coelastrum* [70] but restricted to realized attachment sites, a feature shared with plaque-connected Forteau fossils (cf. figure 1*b,e*). Whatever its phylogenetic proximity to modern groups or the Forteau fossils, *Palaeastrum* suggests that coenobial green algae in general predate the Cambrian appearance of small planar forms. The earliest coenobial fossil record is thus consistent with broader-scale calibrated molecular phylogenies that reconstruct deep Precambrian origins for archaeplastids in non-marine environments, followed by marine radiations of crown-group chlorophytes from the Neoproterozoic [20]. The Forteau assemblage apparently records an extension of this green algal radiation into marine habitats.

### (e) Implications for interpreting Cambrian and Precambrian acritarchs

Having been identified as coenobial green algae, the Forteau fossils cannot be considered acritarchs, which by definition are of unknown affinity [4]. However, some of the poorer Forteau specimens (electronic supplementary material, figures S1 and S2) are only identifiable via better-preserved material. By implication, comparable fossils might have gone unnoticed in other acritarch assemblages. Among the many reported clusters of Cambrian acritarchs, most lack any indication of ordered arrangement or *in vivo* cell attachments [23,24]. However, among specimens assigned to *Synsphaeridium* from the Buen Formation (Cambrian Stage 3–4 of Greenland) are examples with closely packed planar arrangements including a rounded plate with a central cell and seven surrounding, to more *Tapetisphaerites*-like forms, and a *Kahfia*-like ring with additional cells in the lumen (pl. 3 figs. 5–11 of [24], cf. figure 1*m*). Therefore, the Buen ‘*Synsphaeridium*’ should be considered candidate green algal coenobia with links to the Forteau assemblage. Further candidate coenobia include clusters initially assigned to *Asteridium tornatum* (Cambrian Stage 3 of Estonia), occurring in groups of more than 15 cells that overlap in size with those in the Forteau assemblage (5–22 µm) [71]. These specimens have since been reassigned to *Reticella corrugata* and compared instead to prasinophyte algae, with the clusters considered to have arisen by chance [72], although some show ‘stretched’ cell outlines towards contact surfaces (pl. 21 fig. 3, 6 of [71]) justifying the initial interpretation as true colonies [71]. Similarly, clusters of more than 20 interconnected cells of *A. pallidum* (Cambrian Series 2 of Poland) overlap in size and morphology with those in the Forteau assemblage (pl. IV fig. 6 of [73]), as do the planar arrangement of aligned small (*ca* 8 µm) cells and various looser clusters referred to *Synsphaeridium* from the lower Cambrian of Czechia (fig. 2e–g of [74]).

By further implication, how many Cambrian ‘acritarchs’ are in fact the disarticulated components of coenobia? The constituent Forteau cells are markedly varied and should they become disarticulated in life, or during sedimentation, burial or laboratory processing, they might be interpreted as a number of spurious single-cell form-species. Isolated cells with prominent attachment structures should still be recognizable as colonial in origin, so long as they are preserved in a favourable orientation (e.g. figure 2*g* and electronic supplementary material, figure S2*a*), as should cells with ‘stretched’ outlines or an asymmetric distribution of spines (e.g. figure 2*k,l*). However, some of the small, thin-walled constituent cells might be labelled *Asteridium* [73] and the larger cells with sparse thorn-like processes approach the taxon *Globosphaeridium* [71]. Smoother-walled cells would resemble simple leiospheres, or the locally granulate, inclusion-bearing forms of *Archeodiscina* (cf. pl. IV fig. 6 of [73]).

Taxonomy aside, the Forteau coenobia may have had unicellular life history stages or ecological variants. Modern *Scenedesmus* can live as unicells in laboratory cultures [39,75] and an array of unicellular stages are known from hydrodictyaceans including *Pediastrum*, ranging from spherical zygotes to isolated unicells derived from zoospores following early vesicle disintegration [33], and polygonal cells (polyeders) quite unlike the vegetative cells [50]. Even without these hypothetical unicellular forms, the range of variation among the Forteau cells and the opportunity for disarticulation present serious challenges for interpreting Cambrian acritarch diversity.

Various Cambrian acritarchs have been compared previously to green algae, but on very different evidence. In particular, the variable acanthomorph plexus *Skiagia* has been proposed to represent particular green algal life-history stages based on the distribution of internal bodies and excystment structures [22]. Under this model, hypothetical unornamented vegetative stages (potentially represented among *Leiosphaeridia*) would give rise to ornamented resting or reproductive cysts (*Skiagia*). In turn, the large internal body sometimes observed in *Skiagia* would be an endocyst comparable to a green algal zygocyst, released by excystment to give rise to offspring cell(s) or gametes [22]. Similar interpretations have been extended to other Cambrian and Ediacaran acanthomorphic acritarchs [9,17]. Although the Forteau colonies do not include *Skiagia*-like forms and cannot provide a direct test of the hypothesis, they demonstrate that both acanthomorphic ornamentation and dehiscence splits can occur in cells that by analogy with living forms were vegetative structures, not resting cysts. They also contain a range of internal bodies, but modern coenobial analogues do not form endocysts: rather their zygotic cysts arise via external fusion of gametes [33]. The Forteau inclusions may instead represent condensed cell contents [76], preserved subcellular structures [77], or where granular in appearance, perhaps the incipient daughter colonies prior to extrusion into a vesicle (or direct release). In any case, the Forteau assemblage provides a novel line of evidence for green algal representation among Cambrian acritarchs.

### (f) Ecology of colonial phytoplankton

A colonial habit does not imply any particular ecology, and Precambrian fossil colonies (perhaps the richest record) include heterotrophs and autotrophs living in benthic mats [69] or as planktic masses [78]. Modern coenobial colonies represent a special type of developmentally controlled, physically integrated mode of multicellularity known only among non-marine green algae. Besides being photosynthetic, they exhibit a range of ecologies encompassing the flagellate, actively swimming colonies of *Volvox* and relatives [37], and the large (sometimes decimetric) *Hydrodictyon*, which rests benthically or floats in shallow, quiet-water pools [49]. However, modern planar coenobia with robust cell walls (e.g. *Pediastrum* and *Scenedesmus*) are planktic and non-swimming, with adaptations that shed light on the ecology of Cambrian phytoplankton.

Phytoplankton cells must resist uncontrolled sinking in order to maintain some access to the photic zone, achieved most simply by being a very small unicell (e.g. less than 10 µm) [8], which brings further benefits of a large surface area for nutrient uptake, promoting a high growth rate [75]. In larger cells and colonies, the impeding effect of shape on sinking (form resistance) is an important factor [79]. Morphology is not a straightforward predictor of sinking rate, because living cells often use vacuoles or cytoplasm chemistry to regulate their position, and turbulence in the habitat plays a role [79]. Nevertheless, experimental sinking of plastic models confirms the benefit of increased form resistance in colonies with increasing cell number (up to a point) compared with a sphere of equivalent volume [80]. Even so, colonies will always sink faster than if they were separated into their constituent unicells.

A major driver towards larger size in phytoplankton, whether as individual cells or colonies, is to avoid being eaten [8]. However, trade-offs exist here too, because outgrowing the size range vulnerable to protistan microzooplankton can increase the risk of being eaten by larger metazoan mesozooplankton [81]. In general, colonies might be expected to increase effective body size (and thus help evade predation) without incurring the same cost of sinking as a large single cell of equivalent density. There is a wealth of experimental evidence, including from the coenobial green alga *Scenedesmus*, for colony formation as an inducible defence against predators, often via responses to chemical cues in the environment (infochemicals) [81]. For example, laboratory cultures of *Scenedesmus* that exist as unicells in the absence of predators can be induced to form 4-cell and 8-cell colonies by the addition of water filtered from a culture of *Daphnia*, a grazing zooplanktic crustacean [75]. This response is known to reduce grazer clearance rate [81] but carries the cost of a higher rate of sinking for colonies compared to unicells [75]. More generally, additional metabolic or growth-rate costs to colony formation can be expected and may be measurable under sub-optimal light- or nutrient-limiting conditions [81]. Indeed, changes in population structure in cultured *Pediastrum* suggest a trade-off between predation risk from *Daphnia* and nutrient levels (rather than sinking risk) in controlling cell size, colony size and sexual versus asexual life-cycles [82].

Among modern groups of phytoplankton, chain-forming planktic diatoms can increase or reduce the size of an individual colony through cell addition or colony fragmentation in response to predators of different sizes [81]. For example, the presence of grazing copepod crustaceans was found to suppress (not induce) chain formation in *Skeletonema* diatoms, whereas a smaller protistan grazer had no effect on chain length [83]. Coenobial algae lack this level of control because they cannot increase in cell number once formed, and although some coenobia can fragment under certain conditions (e.g. in *Scenedesmus*) [75], variation in cell number can usually only arise during production of daughter coenobia. Longer term, however, the upper limit in typical cell number observed in many coenobial taxa [32] is consistent with an adaptive response to size-selective predation.

A further defence for colonial phytoplankton against predators and sinking is the elaboration of cell surfaces by spines, granules or filaments, which can act to increase effective body size and/or impede handling and thus deter grazing [81]; it can also increase form resistance compared to smooth-walled cells and colonies [80]. Ornamentation, like colony formation, can be induced by predators, for example, in *Scendesmus* where marginal spines are longer in cultures exposed to *Daphnia* [84]. Aside from elaborations of the cell wall, numerous elongate ‘bristles’ up to 200 µm long occur in living coenobial algae including *Pediastrum* [80]; they disappear rapidly upon death and would not be expected to fossilize, but in life they aid suspension and perhaps deter ingestion.

### (g) Ecology of the Cambrian phytoplankton

In the light of the ecologies of modern colonial phytoplankton, the Forteau algae can be interpreted as having a suite of adaptations against sinking and predation. Furthermore, the marked variability in colony form and ornamentation suggests inducible defences varying on ecological timescales. Each round of asexual reproduction had the potential to radically alter the phenotype of the individual, and by extension—if there were common environmental cues—the population. This evolved plasticity would have been a powerful defence against predation by zooplankton, as well as a means of responding to nutrient levels and physical parameters related to sinking. If the Forteau assemblage represents a single biological species, the implied plasticity is impressive even among coenobial taxa, with at least three ways of controlling effective body size: through cell size, cell number and cell wall ornamentation. Non-preservable bristles, if present, would have given further control. The variability in cell number, with frequent departures from 2*^n^*, could imply a relaxed control on cell movement and destination during the early stages of colony formation compared with modern taxa. However, the ability to generate balanced, symmetrical colonies from a wide range of cell numbers could itself be an adaptive trait given the importance of symmetry for resisting sinking and maintaining orientation in the water column [80]. Even if more than one species is represented, the range of adaptations still points to strong selection pressures imposed by predation, perhaps with different colony forms optimized for different prevailing predators and environmental conditions.

The colonial Forteau ‘acritarchs’ reveal the intensity of interactions between phytoplankton and grazing zooplankton by the late early Cambrian. These zooplankton were possibly protistan but more likely metazoan, given the comparable size ranges of colonies developed among living taxa to deter mesozooplanktic crustaceans and rotifers [81]. Such interactions have previously been invoked to account for the comparatively small size and diverse ornamentations among Cambrian acritarchs compared to their larger Neoproterozoic counterparts [8]. Cambrian metazoans with adaptations suitable for handling and processing phytoplankton include small crustaceans with elaborate setal filter plates [85] and mandibles able to process tough cell walls [28]. Direct evidence for metazoan grazing on phytoplankton comes from faecal strings packed with small (*ca* 5 µm) acritarchs [86].

However, very little has been discovered about the phylogenetic composition and ecological strategies of Cambrian (or indeed Palaeozoic) phytoplankton. Secular changes in biomarker composition predict that the eukaryotic fraction of phytoplankton in the Ediacaran and Palaeozoic was dominated by green algae [19], prior to the Mesozoic takeover by ‘red’ plastid lineages [1,2]. Modest morphological support for this early green algal plankton has come from interpretation of certain Palaeozoic palynomorphs as the phycoma stages of prasinophyte green algae [1,17,21], and of *Skiagia* and a suite of comparable acritarchs as stages in green algal life cycles [9,17,22]. In this context, the Forteau algae offer a compelling new example of a green algal lineage in the Cambrian plankton, replete with adaptations against a newly intense grazing pressure from metazoans invading the water column.

At the same time, the broad range of constituent cell morphologies could pose substantial challenges for translating Cambrian acritarch diversity into biological species diversity. The new fossils also demonstrate that robust cell walls, spiny ornamentation and dark internal bodies can occur in vegetative cells rather than resting cysts. Whether or not any pre-Mesozoic acritarchs are actually dinoflagellates—and the evidence is weighted against this [16,17]—the general assumption has been that most acritarchs are the resting cysts of unicellular organisms [6,8,11,21]. To some extent, dinoflagellates provide a useful comparative framework for understanding acritarch variability, diversity and distribution [12–14]. However, the Forteau coenobia give a cautionary reminder that contrasting ecologies were present among eukaryotic phytoplankton in the Cambrian. It remains to be seen whether the rarity of reported Palaeozoic coenobia represents a severe taphonomic filter, or a genuinely minor role in a plankton dominated by unicells (or loosely aggregating colonies). There is also the question of why modern green algal coenobia are restricted to non-marine environments, even though colonial strategies are evidently still important in the marine plankton, notably among chain-forming diatoms. Turnover in Mesozoic phytoplankton composition has been linked to shifting ocean chemistry and differential nutrient use, as well as evolutionary innovations and feedbacks [2,18,87]. The Forteau fossils contribute new evidence to underpin such analyses for deeper time intervals.

## Data Availability

All data (photographic images, stratigraphic details and fossil measurements) are included in the publication, including the electronic supplementary material [88].
